# Dissecting Meta-Analysis in GWAS Era: Bayesian Framework for Gene/Subnetwork-Specific Meta-Analysis

**DOI:** 10.3389/fgene.2022.838518

**Published:** 2022-05-18

**Authors:** Emile R. Chimusa, Joel Defo

**Affiliations:** Division of Human Genetics, Department of Pathology, Institute of Infectious Disease and Molecular Medicine, University of Cape Town, Cape Town, South Africa

**Keywords:** gene, meta-analysis, Bayesian, subnetwork, GWAS

## Abstract

Over the past decades, advanced high-throughput technologies have continuously contributed to genome-wide association studies (GWASs). GWAS meta-analysis has been increasingly adopted, has cross-ancestry replicability, and has power to illuminate the genetic architecture of complex traits, informing about the reliability of estimation effects and their variability across human ancestries. However, detecting genetic variants that have low disease risk still poses a challenge. Designing a meta-analysis approach that combines the effect of various SNPs within genes or genes within pathways from multiple independent population GWASs may be helpful in identifying associations with small effect sizes and increasing the association power. Here, we proposed ancMETA, a Bayesian graph-based framework, to perform the gene/pathway-specific meta-analysis by combining the effect size of multiple SNPs within genes, and genes within subnetwork/pathways across multiple independent population GWASs to deconvolute the interactions between genes underlying the pathogenesis of complex diseases across human populations. We assessed the proposed framework on simulated datasets, and the results show that the proposed model holds promise for increasing statistical power for meta-analysis of genetic variants underlying the pathogenesis of complex diseases. To illustrate the proposed meta-analysis framework, we leverage seven different European bipolar disorder (BD) cohorts, and we identify variants in the angiotensinogen (*AGT*) gene to be significantly associated with BD across all 7 studies. We detect a commonly significant BD-specific subnetwork with the *ESR1* gene as the main hub of a subnetwork, associated with neurotrophin signaling (p = 4*e* ^−14^) and myometrial relaxation and contraction (p = 3*e* ^−08^) pathways. ancMETA provides a new contribution to post-GWAS methodologies and holds promise for comprehensively examining interactions between genes underlying the pathogenesis of genetic diseases and also underlying ethnic differences.

## 1 Introduction

The main goals of trait mapping studies, including genome-wide association studies (GWASs), are to understand the genetic architecture of diseases, pinpoint the number of loci associated with a particular trait, and approximate the underlying heritability rate (Hirschhorn and Daly, 2003; Cantor et al., 2010; Wray et al., 2010; Garfield, 2020). Once the disease-causing variants and genes are identified, this information will help researchers who are working in clinical, medical, or public health fields to establish prevention strategies, predict risks, and adapt therapeutic measurements Hirschhorn and Daly (2003); Cantor et al. (2010); Tam et al. (2019); Nguyen and Eisman (2020); Legge et al. (2021). Regardless of the successes, GWASs are still confronting many challenges and limitations Hao et al., 2019); Zhu et al. (2017) and have received considerable criticism (Zuka et al., 2012; Cantor et al., 2010). The challenges faced by GWASs include 1) the translation of the associated loci into suitable biological hypotheses (Zuka et al., 2012); 2) the issue of missing or hidden heritability (Kang et al., 2010; Hirschhorn and Daly 2003), which has now been partially tackled; 3) the understanding of how multiple modestly associated variants within genes interact to influence a phenotype (Wang et al., 2007; Hao et al., 2019; Tam et al., 2019); 4) the imperfection of asymptotic distribution of the current mixed model association or logistic regression in the specific case of low-frequency variants (Chimusa et al., 2012; Hao et al., 2019); and 5) the inefficiency in distinguishing between inflation from bias (from cryptic relatedness and population stratification) to the true signal from polygenicity (Chimusa et al., 2012; Hao et al., 2019; Tam et al., 2019).

These limitations reflect a gap in our understanding of the mechanisms underlying the pathogenesis of complex traits and diseases. The major source of these shortcomings is the method of GWAS itself as restricted to a single-marker-based testing approach (Wang et al., 2007; Hao et al., 2019; Tam et al., 2019). Various post-GWAS approaches have been proposed to address the single-SNP-based GWAS limitation (Jia et al., 2011; Wu et al., 2009; Wang et al., 2010; Peng et al., 2008), which are different in many aspects, but all are driven by the need to extract useful information from the GWAS summary statistics. The GWAS meta-analysis has become an increasingly adopted method that leverages association summary statistics to fostering a culture of compulsory *in silico* replication to maintain reliability in genetics association findings Ilya et al. (2017); Duarte et al. (2019); Shen and Tseng (2010); Lu et al. (2018). A meta-analysis framework combines results from different GWAS cohorts and puts them in one analysis framework to recover signals that one single GWAS cohort study mighty be missed and address the between-study and between-population heterogeneity Kavvoura and Ioannidis (2008); Thompson et al. (2011). In the last decade, the use of meta-analysis method has increased due to different interests from both the medical researchers and statisticians Shi and Lee (2016); Fan et al. (2016); Turley et al. (2018). Recently, meta-analysis has shown remarkable discovery results and helped to more understand and validate association results from different studies. The meta-analysis is considered as post-genome-wide association study method Chimusa et al. (2012); Han and Eskin (2011); Lu et al. (2018); Shi and Lee (2016). Despite the instrumental findings from single-SNP-based meta-analysis, there remains a need for a single comprehensive analysis that can both aggregate from the diverse population GWAS and incorporate the effect of multiple markers and other potential factors at a gene or pathway level. Heterogeneity among the GWAS meta-analyses remains an issue, particularly when the number of studies increases Han and Eskin (2011); Kavvoura and Ioannidis (2008); Thompson et al. (2011). This raised challenge on the power of GWAS meta-analysis across diverse population cohorts of differing genetics ancestry. Moreover, critical caution is required since incomplete replication can also be informative as several studies reported lack of interpopulation replicability, indicating that some risk variants are population-specific Hirschhorn and Daly (2005); McCarthy et al. (2008); Newton-Cheh and Hirschhorn (2005). For example, comparing the Asian and European associations with major depression, the failure of replication is largely due to the difference in the partner of linkage disequilibrium (LD), which reduces power in one population since the proportion of attributable risk declines with a population-specific minor allele frequency Newton-Cheh and Hirschhorn (2005). A caveat, however, is that fewer GWASs conducted in the non-European ancestry usually constitute of fewer samples Newton-Cheh and Hirschhorn (2005), raising the question as to how the clinical utility of GWASs can be made equitable across multi-ethnic populations Martin et al. (2021); Torkamani et al. (2018) and, specifically, how to accurately predict health and disease risks in the African populations. Furthermore, variation of the cohort size across independent studies is challenging, especially when these studies have been conducted from distinct populations of different ancestries and patterns of LD Chimusa et al. (2012); Han and Eskin (2011); Kavvoura and Ioannidis (2008); Thompson et al. (2011).

While the factors may raise heterogeneity Chimusa et al. (2012); Han and Eskin (2011); Kavvoura and Ioannidis (2008); Thompson et al. (2011), designing a gene-based and subnetwork/pathway-based meta-analysis may be helpful in pooling information from multiple population GWASs and multiple variants within a gene or genes within pathways or subnetworks Shi and Lee (2016); Fan et al. (2016); Turley et al. (2018); Ilya et al. (2017); Duarte et al. (2019); Shen and Tseng (2010); Lu et al. (2018). This may reveal larger effects and provide valuable information to prioritize the most important results across human populations. We refer to this approach as gene- or subnetwork/pathway-specific meta-analysis. Similarly, the list of new post-GWAS tools, such as multi-marker analyses, which go beyond single SNP tests, or the inclusion of functional evidence to reweight GWAS results, is growing by the day Newton-Cheh and Hirschhorn (2005); Chimusa et al. (2012); Shi and Lee (2016); Fan et al. (2016); Turley et al. (2018); Ilya et al. (2017); Duarte et al. (2019); Shen and Tseng (2010); Lu et al. (2018). Although many methods for meta-analysis have been developed over the past decades, the methodology still faces significant limitations. In particular, the challenge of low statistical power is still unresolved, as demonstrated by the fact that meta-analyses have not necessarily resulted in an increased statistical power Li et al. (2012). This is, in part, due to the analysis methods failing to optimally account for the high degree of between-study heterogeneity that characterizes most meta-analyses of the datasets Shi and Lee (2016); Fan et al. (2016); Turley et al. (2018); Duarte et al. (2019); Lu et al. (2018). Apart from power considerations, another important challenge is the translation of statistical association in meta-analyses into biologically meaningful insights.

**FIGURE 1:**
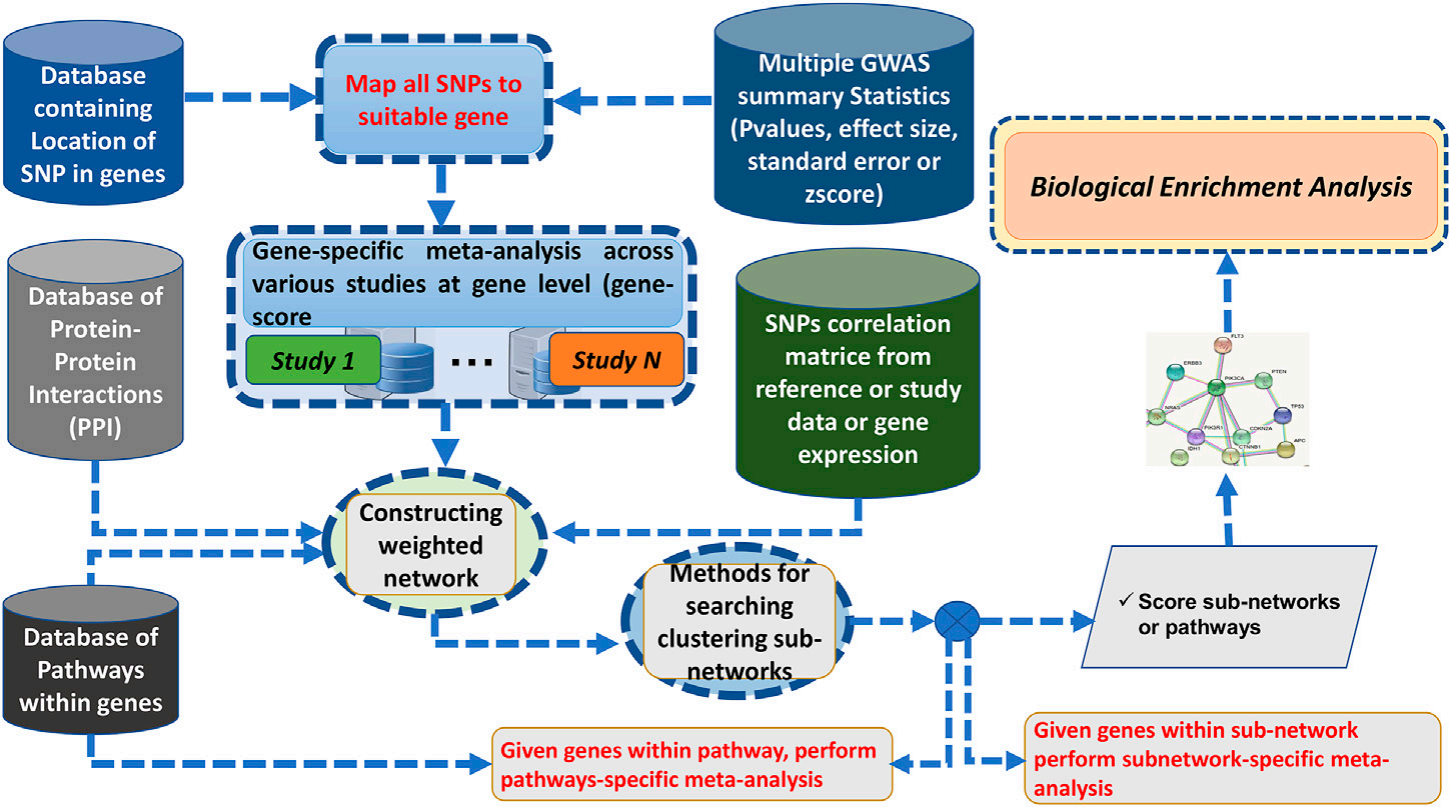
Flowchart of ancMETA.

Here, we addressed some GWAS meta-analysis limitations by proposing a Bayesian graph-based framework to perform the gene/pathway-specific meta-analysis by combining the effect size of multiple SNPs within genes, and genes within subnetwork/pathways across multiple independent population GWAS to deconvolute the interactions between genes underlying the pathogenesis of complex diseases across diverse populations (Figure 1).

We assessed the proposed framework on simulated datasets, and the results show that the proposed model holds promise for increasing statistical power for meta-analysis of genetic variants underlying the pathogenesis of complex diseases. We illustrated it in 7 different European bipolar disorder (BD) cohorts, and we finally outlined the implications, challenges, and opportunities that cross-ancestry meta-analyses present in the GWAS era. The proposed method has been implemented in the ancMETA tool https://github.com/echimusa/ancMETA, providing a new contribution to post-GWAS methodologies, and holds promise for deconvoluting interactions between genes underlying the pathogenesis of genetic diseases and underlying ethnic differences.

## 2 Materials and Methods

### 2.1 Details of Gene/Subnetwork-Specific Meta-Analysis

Here, we discuss the proposed meta-analysis framework, ancMETA. It performs meta-analysis at two different levels by aggregating multiple independent population GWAS summary statistics datasets. It uses an integrative analysis through Bayesian posterior probability and combines the results into known biological protein–protein network datasets. Lastly, ancMETA performs the meta-analysis at the subnetwork level and identifies the most significant subnetworks to understand the biological pathways (Figure 1).

We describe six different steps of the proposed meta-analysis framework as follows:

Step 1: Collection of *N*-independent studies.

This step requires *N* ≥ 2 independent GWAS summary statistics datasets, from the same phenotype or trait. GWAS summary statistics are defined here as per-genetic locus effect sizes (log odds ratios) together with their standard errors, *p*-values, or z-scores for the affected–unaffected traits de Leeuw et al. (2015); Il-Youp and Wei (2015); Huang et al., 2016); Wang et al. (2017).

Step 2: Mapping SNPs to the associated genes.

This is an intermediate step, and it is similar to our previous approach Chimusa et al. (2015), where all the SNPs are mapped to their related genes. It is common practice to assign SNPs to the genes based on a distance cutoff, and the previous studies use a variety of cutoffs, such as distance from 2 to 500 Kbps Conti et al. (2009); Brodie et al. (2016); Chimusa et al. (2015); Wang et al. (2010, 2007). ancMETA allows users to specify the distance cutoffs of assigning SNPs down-/upstream to a specific gene (see the Supplementary Material). At this step, the combined statistical outcomes (i.e., the effect size and the standard error) are computed, as illustrated in Figure 1.

We assume that the *ith* study (*i* = 1, 2,…, *N*) has *J*_*i*_ genes $G_{j}^{i}\;\left(j=1,2,\ldots ,J_{i}\right)$ , each having a set of specific SNPs, where *N* is the total number of studies. Let $S=\cup_{i,j}S_{j}^{i}$ denote the set of all SNPs, where $S_{j}^{i}$ is the set of SNPs at the *jth* gene of the study *i*. For an SNP $s\in S_{j}^{i}$ , let $\beta_{j}^{i}\left(s\right)$ , $\zeta_{j}^{i}\left(s\right)$ , and $p_{j}^{i}\left(s\right)$ represent the effect size, the standard error, and the *p*-value, respectively. The proposed meta-analysis framework assumes that the SNPs related to one gene are correlated. This assumption is due to the fact that each gene contains a large number of SNPs mostly under LD; therefore, the magnitude of the statistical outcomes for each SNP within a gene from each study is considered to be approximately the same. Thus, the effect size and the *p*-value will follow a normal distribution. Let $\mu_{j}^{i}$ be the unknown true effect size at the *jth* gene $G_{j}^{i}$ of study *i* (*i* = 1, 2,…, *N*; *j* = 1, 2,*…*, *J*_*i*_). Thus, a fixed-effects model can be applied to estimate the combined effect size at the gene level from each study *i* = 1, 2, … *N*. We construct a linear function of parameters $\mu_{j}^{i}$ as follows:

$$x=y\left(\beta ,\mu \right)=\underset{s\in S_{j}^{i}}{\sum }\beta_{j}^{i}\left(s\right)\mu_{j}^{i}.$$

Let $V_{j}^{i}\left(s\right)$ be the variance. Then, the estimation of the combined effect size is given as follows:

$$\begin{array}{ccc} p\left(x|B_{j}^{i},\mu_{j}^{i},V_{j}^{i}\right) & & =N\left(x|B_{j}^{i}{\mu_{j}^{i}}^{\;},V_{j}^{i}\right)\\ & & ∼\underset{s\in S_{j}^{i}}{\prod }\frac{1}{\sqrt{2V_{j}^{i}\left(s\right)\pi }}\exp\left[-\frac{{\left(\beta_{j}^{i}\left(s\right)-\mu_{j}^{i}\right)}^{2}}{2V_{j}^{i}\left(s\right)}\right]. \end{array}$$ (1)

Note Eq. 1 is the likelihood when *μ* ≠ 0. Although the solution, which corresponds to the maximum true effect size $\mu_{j}^{i}$ at gene $G_{j}^{i}$ , can be approximated from sampling approaches, Eq. 1 can be solved analytically by differentiating the log likelihood and setting it to 0, that is,

$$\frac{\partial \log\left(p\left(x|B_{j}^{i},\mu_{j}^{i},V_{j}^{i}\right)\right)}{\partial \mu_{j}^{i}}=0$$

or

$$\underset{s\in S_{j}^{i}}{\sum }\left[\frac{\beta_{j}^{i}\left(s\right)-\mu_{j}^{i}}{V_{j}^{i}\left(s\right)}\right]=0.$$ (2)

Eq. 2 can be written as

$$\underset{s\in S_{j}^{i}}{\sum }{\left(V_{j}^{i}\left(s\right)\right)}^{-1}\beta_{j}^{i}\left(s\right)-\underset{s\in S_{j}^{i}}{\sum }{\left(V_{j}^{i}\left(s\right)\right)}^{-1}\mu_{j}^{i}=0.$$

Hence, the estimated effect size of gene $G_{j}^{i}$ is given as

$${\hat{\mu }}_{j}^{i}=\frac{\underset{s\in S_{j}^{i}}{\sum }{\left(V_{j}^{i}\left(s\right)\right)}^{-1}\beta_{j}^{i}\left(s\right)}{\underset{s\in S_{j}^{i}}{\sum }{\left(V_{j}^{i}\left(s\right)\right)}^{-1}}.$$

Therefore, its standard error is derived as

$$sd\left({\hat{\mu }}_{j}^{i}\right)={\left(\sqrt{\underset{s\in S_{j}^{i}}{\sum }V_{j}^{i}\left(s\right)}\right)}^{-1}.$$

Step 3: Meta-analysis at the gene level.

Let $\beta_{j}^{i}$ and $\zeta_{j}^{i}$ be the combined statistical outcomes (i.e., effect size and standard error, respectively). Since the effect size may be different across the studies, the heterogeneity between the studies may be high. A parameter $\sigma_{j}^{2}$ , which accounts for between-study variability, is introduced. Because the maximum-likelihood method underestimates the variance when the number of studies is small DerSimonian and Laird (1986), we use the unbiased method of moments Borenstein et al. (2010) to compute the variance components as follows:

$$\sigma_{j}^{2}=\frac{1}{N}\overset{N}{\underset{i=1}{\sum }}{\left[\beta_{j}^{i}-H_{j}\right]}^{2},$$

where $H_{j}=\frac{1}{N}\sum_{i=1}^{N}\beta_{j}^{i}$ . It is assumed that the true effect size of the gene *G*_*j*_ is $\mu_{j}+\sigma_{j}^{2}$ , where *μ*_*j*_ is an unknown parameter. The same calculations as in step 2 are applied to estimate the effect size of the gene *G*_*j*_ across the studies, which is calculated as follows:

$${\hat{\mu }}_{j}=\frac{\overset{N}{\underset{i=1}{\sum }}{\left(\sigma_{j}^{2}+V_{j}^{i}\right)}^{-1}\beta_{j}^{i}}{\overset{N}{\underset{i=1}{\sum }}{\left(\sigma_{j}^{2}+V_{j}^{i}\right)}^{-1}},$$

and its standard error is given as follows:

$$sd\left({\hat{\mu }}_{j}\right)={\left(\sqrt{\overset{N}{\underset{i=1}{\sum }}\left[\sigma_{j}^{2}+V_{j}^{i}\right]}\right)}^{-1}.$$

If the precision *ρ* ^*i*^ of the study *ith* for *i* = 1, 2,…, *N* is equal to $\frac{1}{{\left(\mu^{i}\right)}^{2}}$ , then the heterogeneity statistic, using Cochrane’s test (*Q*) is given as follows:

$$Q=100\;\times \;\overset{N}{\underset{i}{\sum }}\rho^{i}{\left({\hat{\mu }}_{j}-\theta \right)}^{2},$$ (3)

where $\theta =\frac{\Sigma_{i}^{N}\rho^{i}{\hat{\mu }}_{j}}{\Sigma_{i}^{N}\rho^{i}}$ is a weighted estimator, and $Q∼\chi_{N-1}^{2}$ under the null hypothesis that the between-study variance *τ* ^2^ = 0. If we consider the expectation of Eq. 3, the between-study variance is given as

$$\tau^{2}=\frac{Q-\left(N-1\right)}{\Sigma_{i}^{N}\rho_{i}-\left(\frac{\Sigma_{i}^{N}\rho_{i}^{2}}{\Sigma_{i}^{N}\rho_{i}}\right)}.$$

Since the test statistics in Eq. 3 has N − 1 degree of freedom (*df*), then for *Q* < *df* (which means *τ* ^2^ < 0), the maximum between 0 and *τ* ^2^ is considered, so that *τ* ^2^ is non-negative. Recalling that in step 2, we pointed out that SNPs within one gene are correlated. Therefore, to perform meta-analysis, a fixed-effects model is used Borenstein et al. (2010).

Step 4: Mapping genes to biological networks.

For this step, all the genes and their related statistical outcomes and gene–gene combined LD are mapped into a PPI network database (Supplementary Material), which contains information on interactions among the genes. Therefore, the genes are considered as weighted nodes with their related statistical outcomes (i.e., effect size and standard error), and the edges represent the interactions between the genes (nodes), which are weighted with the combined LD, for each pair-wise genes (nodes) that have a link. More details can be found in Supplementary Materials. The details of the combined LD can be found in Supplementary Text S1.

Step 5: Meta-analysis at the subnetwork level. This step estimates the effect size and standard error of each subnetwork from . Supplementary Text S1 provides more details of different concepts used in . The procedure is the same as that of step 3, where it is assumed that there is a heterogeneity between the genes in each subnetwork. Let $\rho_{j}^{2}$ be the between-gene heterogeneity, $\beta_{j}^{i}$ be the observed effect size, and $\zeta_{j}^{i}$ be its standard error. If *μ* is the unknown true effect size at the subnetwork level with *K* genes, the estimated effect size is given by $p\left(x|\beta_{j}^{i},\mu_{j}^{i},V_{j}^{i}+\rho_{j}^{2}\right)∼$

$$\underset{G_{j}^{i}\in K}{\prod }\frac{1}{\sqrt{2\left(V_{j}^{i}+\rho_{j}^{2}\right)\pi }}e^{\left[-\frac{{\left(\beta_{j}^{i}-\mu_{j}^{i}\right)}^{2}}{2\left(V_{j}^{i}+\rho_{j}^{2}\right)}\right]}.$$ (4)

Since Eq. 4 is considered to be the likelihood when *μ* ≠ 0, maximizing the log likelihood by solving

$$\frac{\partial \log\left(p\left(t|\beta_{j}^{i},\mu ,V_{j}^{i}+\rho_{j}^{2}\right)\right)}{\partial \mu_{j}^{i}}=0$$

yields

$${\hat{\mu }}_{j}^{i}=\frac{\underset{G_{j}^{i}\in K}{\sum }{\left(\rho_{j}^{2}+V_{j}^{i}\right)}^{-1}\beta_{j}}{\underset{G_{j}^{i}\in K}{\sum }{\left(\rho_{j}^{2}+V_{j}^{i}\right)}^{-1}},$$ (5)

and its standard error estimated effect size is given as

$$sd\left({\hat{\mu }}_{j}^{i}\right)={\left(\sqrt{\underset{G_{j}^{i}\in K}{\sum }\left[\rho_{j}^{2}+V_{j}^{i}\right]}\right)}^{-1}.$$

Step 6: Computing the overall statistical significance.

From step 5, we know that $\beta_{j}^{i}$ and $\zeta_{j}^{i}$ represent the observed effect size and the standard error, respectively, where ${\hat{\mu }}_{j}^{i}$ as the unknown effect was estimated using Equation 5. For this step, let us consider the Bayes’ theorem, which includes all the possible effect outcomes for *N* studies, and let us apply the symmetrical property of the Gaussian distribution. Therefore, the posterior probability is approximately given as

$$\begin{array}{ccc} {\hat{e}}_{j}^{i} & & =\frac{\pi \int_{R}N\left(\beta_{j}^{i}|{\hat{\mu }}_{j}^{i},\zeta_{j}^{i}\right)p\left({\hat{\mu }}_{j}^{i}\right)d{\hat{\mu }}_{j}^{i}}{\left(1-\pi \right)N\left(\beta_{j}^{i}|0,\zeta_{j}^{i}\right)+\pi \int_{R}N\left(\beta_{j}^{i}|{\hat{\mu }}_{j}^{i},\zeta_{j}^{i}\right)p\left({\hat{\mu }}_{j}^{i}\right)d{\hat{\mu }}_{j}^{i}}\\ & & =\frac{\pi N\left(\beta_{j}^{i}|{\hat{X}}_{j}^{i},\zeta_{j}^{i}+{\hat{V}}_{j}^{i}\right)}{\left(1-\pi \right)N\left(\mu_{j}^{i}|0,\zeta_{j}^{i}\right)+\pi N\left(\mu_{j}^{i}|{\hat{X}}_{j}^{i},\zeta_{j}^{i}+{\hat{V}}_{j}^{i}\right)}. \end{array}$$ (6)

However, a test statistic based on the weighted sum of chi-square can be used, under the null hypothesis that there is no association. Thus, from the fact that $e_{j}^{i}$ contains the information from all studies, it can be used as a prior weight in the weighted sum of the chi-square statistics as follows:

$${\hat{\chi }}_{j}=\frac{\overset{N}{\underset{i=1}{\sum }}{\hat{e}}_{j}^{i}}{\sqrt{{\hat{e}}_{j}^{i}}}\frac{{\left({\hat{\mu }}_{j}\right)}^{2}}{\left(\sum_{i=1}^{N}\zeta_{j}^{i}+\tau^{2}\right)},\;\tau^{2}\geq 0,$$

where *τ* ^2^ is the between-study variance. Recalling that Equation 6 is not normally distributed, the *p*-values can be evaluated through a sampling method developed by Han and Eskin (2011). puts together all the steps of the proposed framework.

**Table:** 

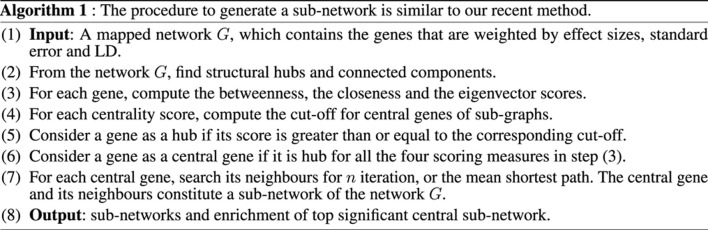

**Table:** 

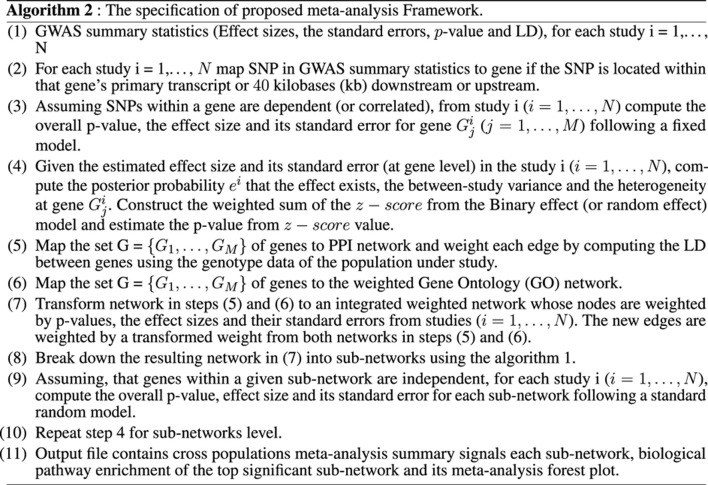

### 2.2 Evaluation of ancMETA From Simulation GWAS Data

The U.S. residents of northern and western European ancestry (CEU), Yoruba (YRI), and Mexican (MEX) populations from the HapMap3 Project were used to generate independent case–control studies. Details of these populations can be found in Supplementary Table S1. We independently performed a population growth model on each dataset mentioned previously (Supplementary Text S2). From the resulting expanded datasets, we generated three independent case–control datasets based on chromosomes 1 and 22 using HapGen2 Su et al. (2011). Here, we randomly selected 3 SNPs on chromosome 1 and 3 other SNPs on chromosome 22 to be simulated as causal disease SNPs with differing effect sizes under HapGen2 Su et al. (2011). The simulated disease effect size parameters of those SNPs are summarized in Supplementary Table S2. These parameters are chosen to fit small effect size in some studies and strong in others. We simulated 1,000, 3,000, and 950 cases and 1,000, 3,000, and 1,000 controls from the haplotype (combination of multiple SNPs) data of CEU, YRI, and MEX, respectively. We considered the simulated MEX GWAS dataset as our primary study. The simulation details can be found in Supplementary Text S2.

We conducted a GWAS on each dataset using EMMAX Kang et al. (2010). As expected and according to our simulation parameters (Supplementary Table S2), the GWAS results indicate significant, moderate, and weak signals of association in CEU, YRI, and MEX, respectively, as per simulation (Supplementary Table S3 and Supplementary Figure S1). We used the resulting GWAS summary statistics as input in our proposed model, implemented in “ancMETA,” to perform the gene- and subnetwork-specific meta-analysis (Supplementary Text S3).

**FIGURE 2:**
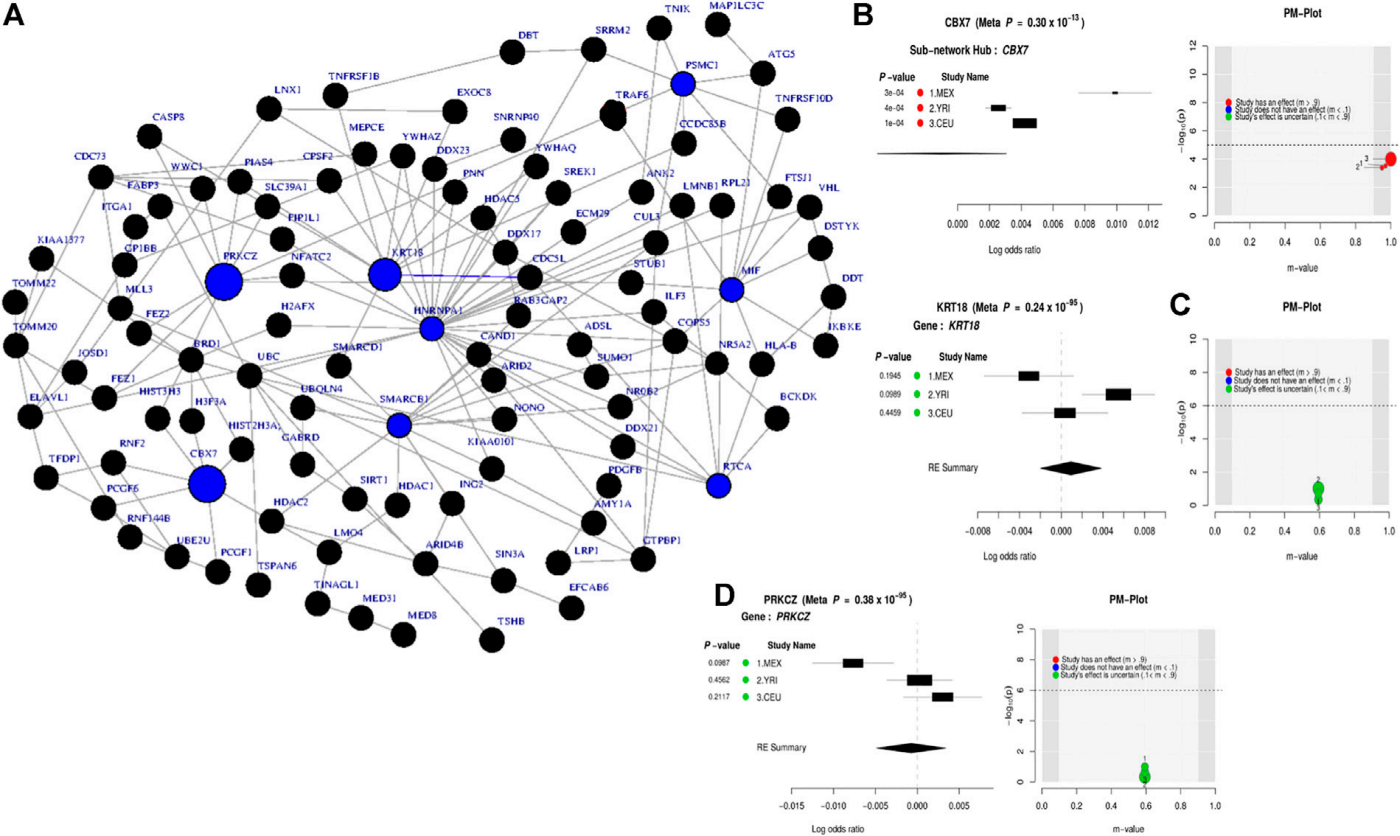
Combined statistical outcomes at the subnetwork level, explaining causality relationship between the simulated phenotype and population variation population based on 3 independent simulated GWAS datasets from CEU, YRI, and MEX, respectively. **(A)** Top significant meta-analysis-based sub-network from ancMETA where nodes in blue colour denote critical hub and genes associated to the simulated causal SNPs. The size of a node denotes its statistical significance from small to large. **(B-D)** Forest plot of three gene-hub from the top 3 meta-analysis sub-networks produced from ancMETA.

At the gene level, the results in Table 1 and Supplementary Figure S2 show significant genes, including *CBX7*, *LD0C1L*, and *ASTN1* associated with our simulated causal SNPs (Supplementary Table S2). This result indicates further increase in the effect size across these 3 simulated case–control studies, compared to single-SNP tests in GWAS based on EMMAX (Supplementary Table S3). Interestingly, at a subnetwork level, we observed a significant convergence of effect sizes of the simulated disease variants across all the studies (Figures 2A,B), particularly to *CBX7* (Table 1). This supports the fact that the true effect risk-associated variants may differ across populations at the polymorphism level, but the effect may tend to convert at a similar magnitude at gene and pathway levels or overlap in the same biological subnetworks/pathways when aggregating several small polymorphism effects.

**TABLE 1:** Meta-analysis at gene and subnetwork levels from the simulated GWAS summary statistics across 3 studies: results show convergence of effects across studies to the simulated causal variants.

					Study p-values			Study M-values		
Gene	#Study	P	Q	Tau square	MEX	YRI	CEU	MEX	YRI	CEU
*LDOC1L*	3	1.33e-10	2.055	0.0007	0.012	0.00019	0.0003	0.98	0.91	0.81
*PRKCZ*	3	1.77e-11	2.193	0.001	0.001	0.047	0.001	0.40	0.41	0.41
*CBX7*	3	0.00000001	1.825	0	0.0001	0.001	0.0001	0.90	0.91	0.90
*ASTN1*	3	2.006e-8	2.261	0.001	0.0012	0.001	0.0006	0.81	0.91	0.91
**Hub**	**#Study**	**P**	**Q**	**Tau Square**	**MEX**	**YRI**	**CEU**	**MEX**	**YRI**	**CEU**
*CBX7*	3	4.25e-7	2.1	0.0002	0.0003	0.0004	0.0001	0.98	0.96	1.0
*PRKCZ*	3	1.93e-7	2.2	0.0002	0.019	0.04	0.021	0.59371	0.59	0.59
*HNRNPA1*	3	3e-08	2.09	0.00008	0.09	0.048	0.03	0.59	0.59	0.59
*KRT18*	3	4e-07	1.90	0	0.049	0.09	0.005	0.59	0.59	0.59

### 2.3 Application to Seven European With Bipolar Disorder Cohorts

Here, we used seven European bipolar disorder (BD) GWAS summary statistics obtained from the NIMH data repository (Supplementary Table S1). These datasets include Irish (IRI), Scottish (SCT), 3 European American (EUA), Norwegian (NOR), and British (BRB) individuals. Supplementary Table S4 provides GWAS summary results of each of these studies. Leveraging the European reference panel from 1000 Genomes Project data, we first conducted the imputation LD fine-mapping from the obtained GWAS summary statistics using ImpG-Summary Pasaniuc et al. (1977) to possibly unravel more LD-SNP association (Supplementary Figure S3). The resulting imputation GWAS LD fine-mapping summary statistics were used as input for ancMETA. We performed the meta-analysis at a gene level across these 7 studies, and seven genes were identified (*AGT CACNA1C*, *ESR1*, *NCAN*, *BDNF*, *BCR*, and *GSK3B*), of which *AGT* has a strong effect across the European populations including Irish, Scottish, Norwegian, and British in contrast to the European American (Supplementary Figures S4,5 and Supplementary Table S5).

**FIGURE 3:**
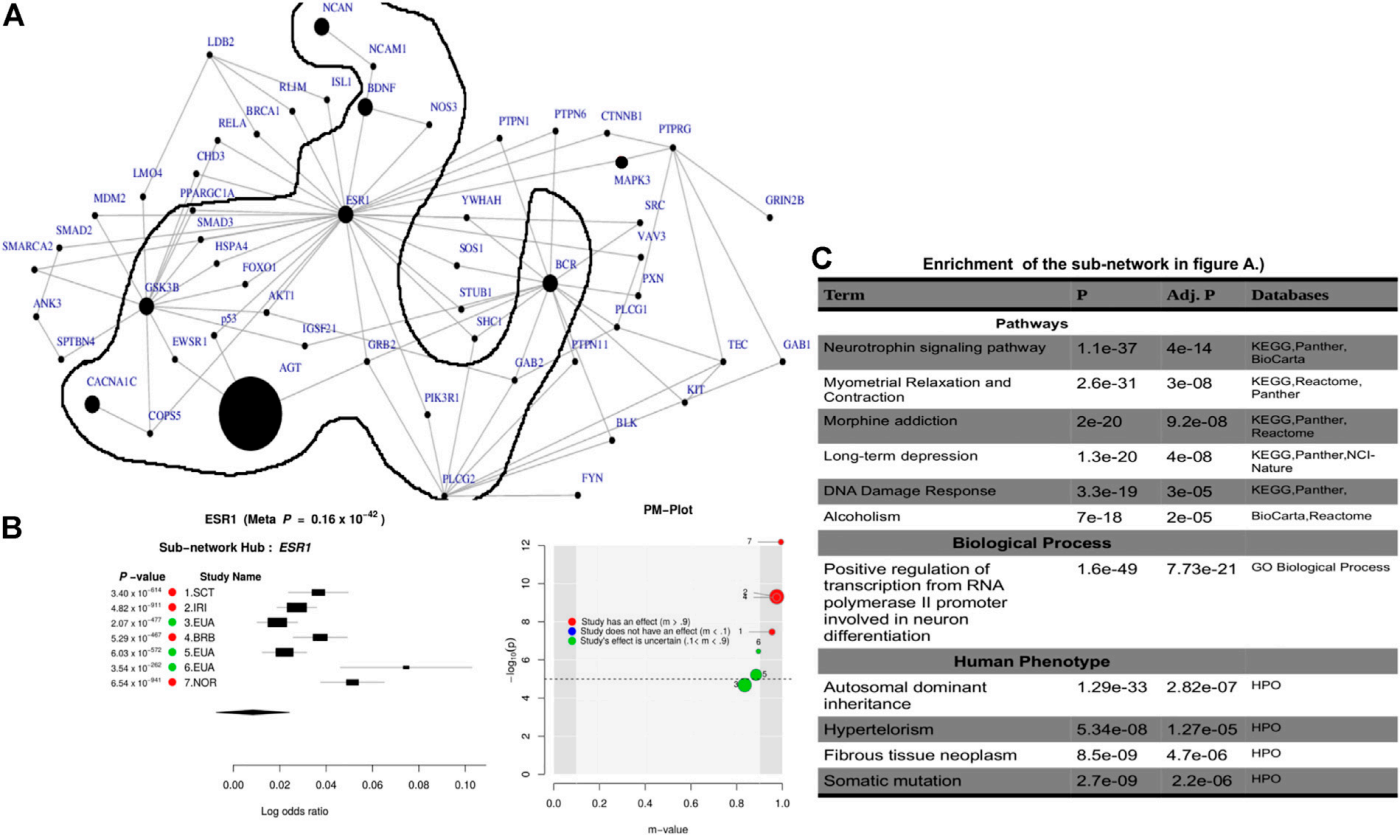
Combined statistical outcomes at the subnetwork level, explaining causality relationship between the simulated phenotype and population variation based on 7 independent GWAS datasets from European bipolar cohorts. **(A)** The top significant meta-analysis-based sub-network where the size of a node denotes its statistical significance from small to large and large sized node are associated genes or genes interacting with known Bipolar genes. **(B)** Forest plot of the gene-hub from the top meta-analysis sub-networks in **(A, C)** Enrichment analysi based on pathways, biological process and human phenotype associated to the top significant meta-analysis-based sub-network in **(A)**.

Leveraging the recent version of the human PPI network (IntAct release 239) from the IntAct database Kerrien et al. (2012); Orchard et al. (2014), we observed a significant connected subnetwork where the estrogen receptor 1 (*ESR*1) gene is the main hub. *ESR1* interacts with *AGT* and is connected with the other gene hubs with known BD-associated genes such as *CACNA1C*, *PLCG2*, *NCAN*, *BCR*, and *BDNF*. Supplementary Table S5 summarizes the association of the top subnetwork across these 7 studies. The identified subnetwork (Figures 3A,B) is significantly associated with the neurotrophin signaling (p = 4*e* ^−14^) and other interesting biological pathways such as myometrial relaxation and contraction (p = 3*e* ^−08^), morphine addiction (*p* = 9.2*e* ^−08^), DNA damage response (p = 3*e* ^−05^), and alcoholism (p = 2*e* ^−05^). In addition, the subnetwork (Figure 3C) is implicated in a positive regulation of transcription from RNA polymerase II promoter involved in neuronal differentiation (*p* = 7.7*e* ^−21^) and is also associated with autosomal dominant (*p* = 2.8*e* ^−07^) inheritance types of diseases (Figure 3C).

**FIGURE 4:**
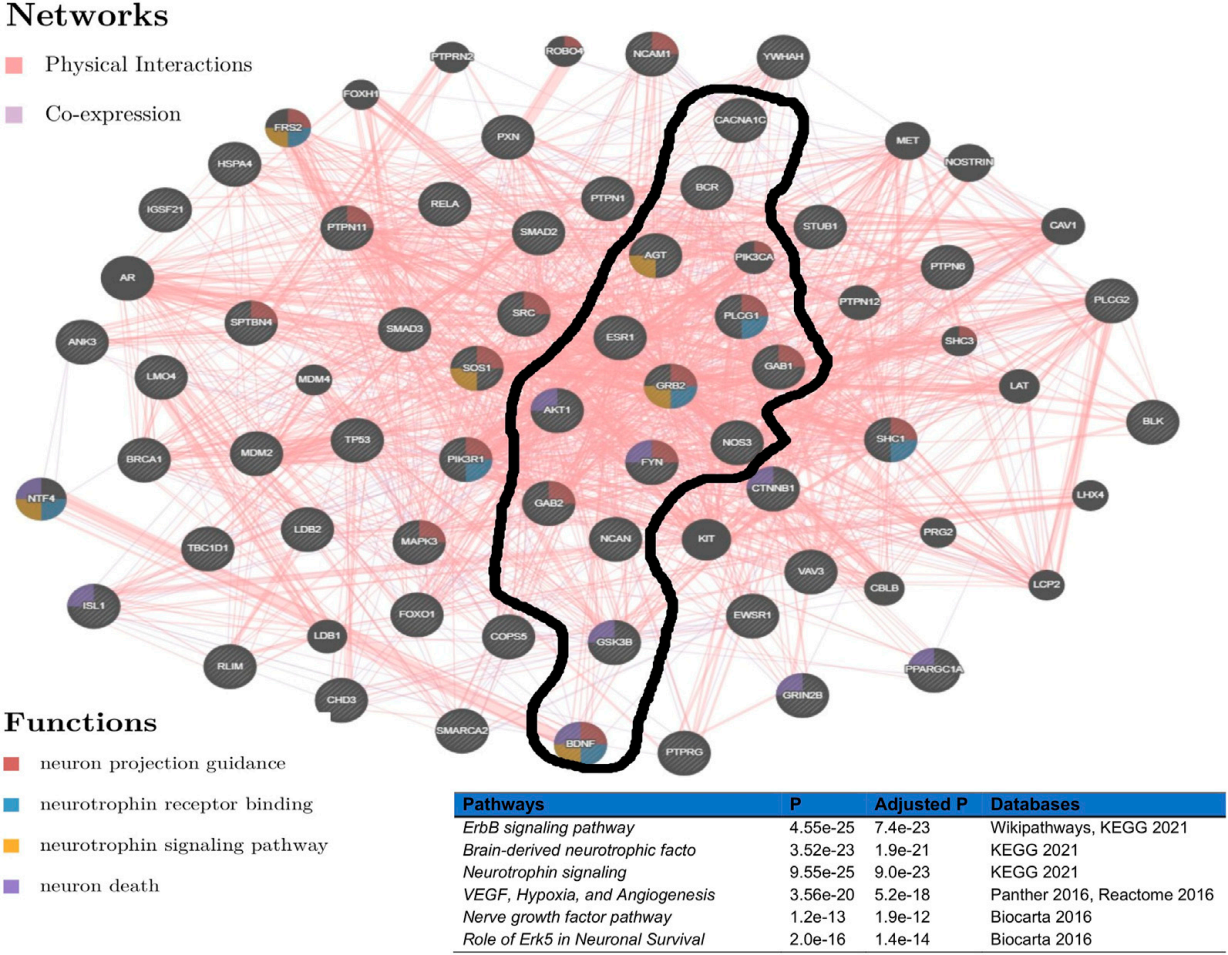
Network reconstructed from genes in Figure 3, based on physical and co-expressed interactions obtained GeneMania Franz et al. (2018) and the top pathways of the reconstructed network from Figure 3 obtained from various pathways databases in Enrichr Kuleshov et al. (2016).

To evaluate the determined interconnectivity among *AGT*, *CACNA1C*, *ESR1*, *NCAN*, *BDNF*, *BCR*, and *GSK3B* as well as with the other protein-coding genes in Figure 3, and their association with BD, we leverage GeneMania Franz et al. (2018) to reconstruct the network shown in Figure 3 based on physical and co-expressed interactions. The result from the reconstructed co-expression and physical interaction network of genes in Figure 3A confirms that the above 7 genes detected by ancMETA (Figure 3A) are physically and co-expressly interconnected Figure 4 and as well as with other protein-coding genes in Figure 3A. From the enrichment analysis based on Enrichr Kuleshov et al. (2016), the network in Figure 4 is significantly associated with neurotrophin signaling and other interesting biological pathways such as ErbB signaling pathways (Figure 4). Interestingly, recent studies have revealed that complex ErbB signaling networks regulate the assembly of neural circuitry, myelination, neurotransmission, and synaptic plasticity Mei and Nave (2014). Evidence indicates that there is an optimal level of ErbB signaling in the brain, and a deviation from it impairs brain functions. The ErbB signaling pathway may provide therapeutic targets for specific neuropsychiatric symptoms, and dysregulation in the ErbB signaling pathway may explain abnormalities of neural precursor migration in BD Mei and Nave (2014).

## 3 Discussion and Conclusion

Designing a post-GWAS meta-analysis that leverages the combined effect of multiple SNPs within a gene or genes within subnetworks/pathways across multiple GWAS datasets may reveal consensus association signals and identify large effect sizes. This may further provide valuable information in prioritizing the most important results across different populations. Here, we proposed a Bayesian graph-based gene- and pathway-specific meta-analysis approach. We implemented the proposed model in ancMETA (Figure 1), which addresses the variation in effect size across several independent GWAS summary statistics from distinct populations of different ancestry background. We assessed ancMETA through the simulation of different ancestries in three different GWAS studies.

A well-reconstructed human protein–protein interaction network is a powerful tool in network biology and medicine research, which forms the basis for multi-omics and dynamic analyses Mortezaei and Tavallaei (2020). However, the topology of the network and their connectivity may be very sensible from various pathway analysis methods Mazandu and Mulder (2011) in reflecting the relationship between certain biological processes or densely connected multi-protein complexes of biological relevance Franz et al. (2018); Kuleshov et al. (2016). This makes it challenging to compare the different post-GWAS pathway-based or network-based methods Jia et al. (2011); Chimusa et al. (2015); Wang et al. (2015); Mishra and Macgregor (2015); Huang et al., 2016; Wang et al. (2017); de Leeuw et al. (2015).

In addition to the GWAS summary statistics, ancMETA allows users to use any weighted biological network and accepts a user-defined network. In case if users provide an unweighted biological network, ancMETA leverages case–control genotype datasets to construct the weights of the network (Supplementary Text S1), in which the current post-GWAS approaches Fan et al. (2016); Turley et al. (2018); Ilya et al. (2017); Duarte et al. (2019); Shen and Tseng (2010); Lu et al. (2018); Mishra and Macgregor (2015); Huang et al., 2016; Wang et al. (2017); de Leeuw et al. (2015); Jia et al. (2011); Wang et al. (2015) do not account for. Evidence shows that many disorders are “polygenic” (many genetic loci contribute to risk) and reflect disruptions in proteins that participate and involve complex interactions between genes Dudbridge (2016); Khera et al. (2018). In contrast to other tools Shi and Lee (2016); Fan et al. (2016); Turley et al. (2018); Ilya et al. (2017); Duarte et al. (2019); Shen and Tseng (2010); Lu et al. (2018), ancMETA leverages the advantage of topological properties of biological networks to ascertain the interaction of proteins/genes that can be involved in a pathway. Our method accounts for the correlation that exists between the SNPs within a gene or genes within pathways and introduces flexibility in estimating the gene-specific and subnetwork-specific effect size, which, to our knowledge, is a new contribution to post-GWAS methodologies. The proposed framework holds promise for comprehensively examining the interactions between genes underlying the pathogenesis of genetic diseases.

Some improvements need to be considered in future work, such as accurately modeling the convergence of the SNP signal to the related subnetworks and leveraging the weakly/moderately associated signals from different GWAS studies. It is worth mentioning that we have applied ancMETA on old BD GWAS with a limited sample size (see Supplementary Table S5). However, regardless of the trait or phenotype used by the user in using ancMETA, the validity of the outcome will benefit and be improved by employing the powered GWAS summary statistics from GWAS datasets associated with larger numbers of samples (cases/controls).

There is also a need to integrate summary-level data across multiple phenotypes to simultaneously capture the evidence of the aggregate-level pleiotropic association. The lack of accurate knowledge of complex traits and the sensitivity of human protein interaction network makes it challenging to directly compare the results from the different pathway analysis methods. Overall, the method implemented in the proposed framework highlights the value of identifying the effect size of pathways associated with a disease, which may be useful in understanding the pathogenesis, disease risk prediction, and susceptibility to genetic diseases.

The results obtained from the GWAS summary statistics on the European BD cohorts found association with seven genes, of which *CACNA1C* and *NCAN* have previously been implicated in BD through GWAS Bellantuono et al. (2007); Ferreira et al. (2008); Gordovez and McMahon (2020); O’Connell and Coombes (2021). This meta-analysis therefore strengthens these initial results and demonstrates that ancMETA can successfully validate GWAS findings. Interestingly, *AGT* has not reached GWAS significance in the previous studies despite being considered as a candidate in BD due to its role in the renin–angiotensin system Ferreira et al. (2008); Gordovez and McMahon (2020); O’Connell and Coombes (2021). The high significance for *AGT* from this analysis (*p* = 3.2*e* ^−19^) therefore strengthens its association with BD and further highlights the potential impact of ancMETA as a useful tool in discovering additional small effect variants that may be missed in the single GWAS. Importantly, the very interesting *AGT* finding is due to the innovations made in addressing population variation and to the inclusion of a PPI framework. The results from the subnetwork analysis revealed a strong interaction of the hub gene *ESR1* with *AGT*, as well as *CACNA1C*, *NCAN*, *BCR*, *GSK3B,* and *BDNF*, suggesting the fact that the *AGT* finding here is not merely indicative of a false positive, but rather that is valid. In addition to a moderate link to BD, *ESR1* has been related to migraine onset, alcohol dependence, obsessive compulsive disorder, and postpartum depression Alonso et al. (2011). The pleiotropy and genetic overlap between BD and these and other psychiatric phenotypes is a considerable complex Carmiol et al. (2014), suggesting that this analysis may have identified a key hub network and genetic underpinnings in not only BD etiology but also across several psychiatric phenotypes.

## Data Availability

The original contributions presented in the study are included in the article/Supplementary Material, Further inquiries can be directed to the corresponding author.
