# Supplementary material for: Dissecting Meta-Analysis in GWAS Era: Bayesian Framework for Gene/Subnetwork-Specific Meta-Analysis
Source: Front Genet. 2022 May 18;13:838518. doi: 10.3389/fgene.2022.838518 (PMC9159898; doi:10.3389/fgene.2022.838518)
Supplement: Supplementary file 1 [file DataSheet1.PDF]

Supplementary Materials

1 SUPPLEMENTARY DATA

To evaluate our developed tool, we extracted three populations from HapMap 3 data sets `http://www.sanger.ac.uk/resources/downloads/human/hapmap3.html`. In each of the simulation we used whole-genome data of $1,604,948$ overlap SNPs, from the HapMap3 of 165 northern and western Europe (CEU), 203 Yoruba in Ibadan, Nigeria (YRI) and 86 Mexican ancestry in Los Angeles, California samples (see Table S1). To apply our developed tool, we used European population with Bipolar disorder (BD) GWAS datasets, with which we have been granted access, which are stored at the NIMH data repository. BD datasets comprised of seven different studies, with $3,793$ cases and $3,440$ controls in total (see Table S1).

2 SUPPLEMENTARY METHODS

We implemented the proposed model, called ancMETA using python programming language and made it available for public via this link (`https://github.com/echimusa/ancMETA`). Implemented module related functionalities to map the associated signal into protein-protein network and visualize the results of network statistics, are updated from our previous model Chimusa et al. (2015). The protein-protein interaction (PPI) dataset was manually updated from our previous method Chimusa et al. (2015), initially based on KEGG, BioCarta and Ambion GeneAssist Pathway Atlas pathway databases and from the recent version of the human protein-protein interaction (PPI) network (IntAct release 239) from IntAct database (Kerrien et al., 2012; Orchard et al., 2014). A topological test was performed on the constructed LD-weighted network of 21,429 pair-wise PPI network. The annotated human pathways was obtained from Zhang et al. (2012) and collected more from annotated pathways include the KEGG, BioCarta and Ambion GeneAssist Pathway Atlas pathway databases. We downloaded genomic coordinates for all genes from the NCBI ftp-server `ftp://ftp.ncbi.nih.gov`. All these data sets come along with ancMETA

2.1 Text S1:Leveraging the topological properties of network in searching clustered sub-networks

2.1.1 Searching for Sub-networks using Centrality Measures

Given two different sets of SNPs $S^a = \{s_i\}_{i=1,2,\ldots,m}$ and $S^b = \{s_j\}_{i=1,2,\ldots,n}$, ($s_i \neq s_j$, $i = 1, 2, \ldots, m$ and $j = 1, 2, \ldots, n$) associated with genes G_a and G_b, the pair-wise LD between SNPs in S^a and S^b are computed using the r^2 measure Kristin et al. (2002) to get a combined LD. We provide two approaches for weighting (estimating the combined LD at the gene level) the interaction between genes in the Protein-Protein interaction network.

Case 1: Assuming sets of SNPs $S^a = \{s_i\}_{i=1,2,\ldots,m}$ and $S^b = \{s_j\}_{j=1,2,\ldots,n}$, $s_i \neq s_j$, for $i = 1, 2, \ldots, m$, and for $j = 1, 2, \ldots, n$ are assigned to genes G_a and G_b; and the pairwise LD of SNPs between G_a and G_b are independent. Because the distribution of the LD is not normal, thus from ($s_i \neq s_j$) we compute the average z-transforms of LD from all possible combination of pairs of SNPs between genes G_a and G_b. The z-transforms of LD are normally distributed with mean 0 and variance 1 Choi (1977). We compute the combined LD between two genes G_a and G_b as follows,

$$r_{G_aG_b} = tanh\left(\frac{\sum_{i\neq j}^{N} tanh^{-1}\left(LD_{s_is_j}\right)}{N}\right). \quad \text{(S1)}$$

Case 2: Alternatively, if SNPs between a given pair of genes are dependent or correlated, we consider the maximum $r_{G_aG_b} = tanh(\max_{i\neq j}(tanh^{-1}\left(LD_{s_is_j}\right)))$ among all possible N pairs of SNPs between the given pair of genes.

The combined LD is used as the weight of the edge between G_a and G_b genes in the PPI network.

2.1.2 Searching for Sub-networks using Centrality Measures

Considering a weighted Protein-Protein interaction (PPI) network as an undirected network, $G = (V, E)$, where V is the set of n genes as nodes and E is the set of edges as interactions found between genes weighted using gene-correlation. To cluster G into sub-networks, we analyse the general properties of G and quantify the usefulness of each gene in G using their centrality scores; closeness, betweenness, degree or eigenvector. Let us first define the following centrality measures,

(1) **Degree Centrality:**
The degree centrality C_d of a gene in an undirected graph G is the number of genes in the network interacting with it.

(2) **Closeness Centrality Measure:**
The closeness centrality $C_{c(u)}$ of a node u in a network is the inverse of the average distance to all other nodes connected to it Mazandu and Mulder (2011), i.e., $C_{c(u)} = \frac{1}{\frac{1}{m}\sum_{v\in V_u} dist(u,v)}$, where V_u is the set of nodes connected to u, $m = |V_u|$ the number of nodes in V_u and $dist(u, v)$ is the shortest communication path between u and v in the network.

(3) **Communication Betweenness Centrality Measure:**
Let γ_{uv} be the number of communications between genes u and v, and $\gamma_{uv}\,(t)$ the number of shortest communications between u and v in the network G using t as an interior node, for t, u, $v \in V\,(G)$. The rate of communication between u and v, Δ_{uv} that can be controlled by an interior gene t, is given by

$$\Delta_{uv}(t) = \frac{\gamma_{uv}\,(t)}{\gamma_{uv}},$$

if $\gamma_{uv} = 0$, then we set $\Delta_{uv} = 0$. The shortest communication betweenness centrality $C_{spb}\,(t)$ is given by

$$C_{spb} = \sum_{u\in V\wedge u\neq t}\;\sum_{v\in V\wedge v\neq t} \Delta_{uv}\,(t)\,.$$

(4) **Eigenvector Centrality Measure:**
The eigenvector centrality measure assigns relative weights to all genes in the network based on the fact that connections to high-weighted genes contribute more to the weight of the gene target. This means the weight or the contribution x_u of the gene u to the functioning of the system is proportional to the sum of the scores of all genes v connected to u, i.e.,

$$\sum_{(u,v)\in E} x_v = \lambda x_u$$

where λ is a constant of proportionality and x_z denotes the contribution of gene z.
In terms of the adjacency matrix $\mathcal{A} = (a_{uv})_{1\leq u,v\leq n}$, we have

$$\sum_{v=1}^{n} a_{uv} x_v = \lambda x_u$$

with n the number of genes in the network. It turns out that λ is simply an eigenvalue of the adjacency matrix and the vector of contributions of genes is the eigenvector associated with λ.

Let $o(G)$ be the order, $s(G)$ the size of G and SP_{mean}, the mean shortest-communication from every node or gene to every destination within the network G.

Note that the cut-off of different network centrality measures are estimated using general topological properties of the network Mazandu and Mulder (2011). For the betweenness measure, the cut-off is the total number of shortest paths expected in the network, which is approximately $o(G) * SP_{mean}$. For the closeness metric, as defined above, the cut-off is $1/SP_{mean}$ and that of the degree centrality measure is the number of expected interacting genes of a gene in the network, which is $s(G)/o(G)$. In the case of the eigenvector measure, the cut-off is the mean value of the weight or contribution vector of all genes in the network.

(1) Given network G, find structural hubs and connected components;
(2) For each gene, compute the betweenness, the closeness and the eigenvector scores;
(3) For each centrality score, compute the cut-off for central genes of sub-graphs BetOf, ClosOf, DegOf and EigOf;
(4) Consider a gene as a hub if its score is greater than or equal to the corresponding cut-off;
(5) Consider a gene as a central gene if it is hub for all the four scoring measures in step (3);
(6) For each central gene, search its neighbours given a step n or the mean shortest path. The central gene and its neighbours constitute a sub-network of G.

We perform the steps above to identify sub-networks using centrality scores of each gene.

2.1.3 Multiple Correction Procedure

To account for the influence of possible type I error and account for gene/sub-network difference on the number of associated SNPs within and between studies statistics of significance, we apply a similar algorithm to the Benjamini-Hochberg Benjamini and Hochberg (1995) false-discovery correction method on their summary statistics.

(1) Rank the p-value of each gene/sub-network in order from the smallest to the largest.
(2) Multiply the largest p-value by the number of genes/sub-networks in test.
(3) Take the second largest p-value and multiply it by the total number of genes/sub-networks in gene/sub-network list divided by its rank. If less than 0.05, it is significant. Corrected p-value = p-value$* \left(\frac{n}{n-1}\right) < 0.05$, if so, gene is significant.
(4) Take the third p-value and proceed as in step 3: Corrected p-value = p-value$* \left(\frac{n}{n-2}\right) < 0.05$, if so, gene/sub-network is significant.
(5) Repeat step (1) to (4).

Below, we will discuss on how to characterize enriched sub-network given the trait ender study.

2.1.4 Characterization of enriched sub-networks

Here we aim to identify the association between sub-network (obtained from our network-based clustering approach). Let S_i, $(i = 1, \ldots, T)$ within $n_1, \ldots, n_T$ genes and human pathway $P_j \in \mathcal{P}$ be the set of human pathways. We obtained annotated pathways from Zhang et al. (2012) and collected more from annotated pathways include the KEGG, BioCarta and Ambion GeneAssist Pathway Atlas pathway databases. We downloaded genomic coordinates for all genes from the NCBI ftp-server `ftp://ftp.ncbi.nih.gov`

and retained only entries for the human reference sequence. We assign the SNPs located within a gene or less than $40kb$ distance up/downstream of the gene. Let α be the intersection between genes within S_i and genes within pathway P_j. Let β be the intersection between genes within S_i and the total genes in $P_{j,(j=1,...,J)}$. Let N^* be the intersection between genes in the P_j pathway and the total genes in $P_{j,(j=1,...,J)}$, and M^* be the total genes in $P_{j,(j=1,...,J)}$. We compute the statistic of significance of overlap between sub-network S_i of n_t genes and a given pathway P_j using the z-score (A_S), which employs the binomial proportions test Berger et al. (2007),

$$A_S = \left(\frac{\alpha}{N^*} - \frac{\beta}{M^*}\right) \Big/ \sqrt{\frac{\frac{\beta}{M^*}.\left(1 - \frac{\beta}{M^*}\right)}{M^*}} \quad \text{(S2)}$$

2.2 Text S2: Evaluation of the designed method though simulation

The simulation data was generated using a simulation algorithm in HapGen2 Su et al. (2011). We simulated three independent case-control studies using CEU, YRI and MEX (see Table 1) by extraction data of chromosome 1 and 22 from HapMap3 Project Frazer and et al (2007); McVean et al. (2012).

For each datasets, we further conducted quality control to remove all structured, indel, multi-allelic variants and those with low minor allele frequency (MAF $<$ 0.05) prior rephasing. We first phased and inferred the haplotypes using Eagle Loh et al. (2016) from the resulting curated data in Table 1. We performed a post-phasing quality control. We checked the switch-error between haplotypes panel and curated 1000 Genomes haplotypes McVean et al. (2012), 99.7%, 98.05%, 97.05% of sites were with no phase switch-error in these 3 panels (CEU, YRI an MEX), respectively. We further compared sites discordance between these haplotypes panels and independently with their original VCF file prior phasing. Only sites with phase switch error showed discrepancies in MAF, therefore were removed.

Prior to simulation, we independently expanded each haplotype panel following Rogers and Harpendings (1992) model of exponential population growth Williams (1997); Li and Stephens (2003). We implemented this model using three parameters $a_0 = 2P_0\mu$, $a_1 = 2P_1\mu$ and $\lambda = 2\mu t$ where P_0 which is the population size of an initial population assuming to grow exponentially to a new population size P_1 at a time t generation back from present. The mutation rate μ, is the per-generation probability that a mutation strikes a random nucleotide along the genome. Each panel was expanded to $2,000$ haplotypes.

To simulated case-control data set from each haplotype panel, we used Hapgen2 Su et al. (2011). Hapgen2 simulator applies alternative resampling approach to simulate case/control samples. It also has the ability to simulate data for many populations. In addition, it allows the user to specify risk allele, heterozygote and homozygote relative risks. Hapgen2 simulator uses Li and Stephens (LS) model of LD Li and Stephens (2003), by considering reference panel of haplotypes say $T^P = T_1, T_2, \dots T_P$ as input, where each haplotypes is located at B biallelic sites. The LS model takes each new haplotypes as a deficient mosiac of the haplotypes T^P. Controls are simulated by adding haplotypes under LD model and by using copy states, which are developed through Markov ways. The haplotypes are simulated into three stages. Firstly, the cross-over events (by using transition probability) mimic the effect of recombination and separate them into independent segments. Secondly, the copying state is uniformly sampled, and lastly, the allele at each SNP is conditionally simulated on the copying state and a mutation parameter. Cases are simulated in the same way, but in pairs, hence the simulation of each haplotype pair arise from four steps.

Here, we simulated $1,000$, $3,000$, 950 cases and $1,000$, $3,000$, $1,000$ controls from the haplotype (combination of multiple SNPs) data of CEU, YRI and MEX respectively. We randomly selected 3 SNPs on chromosome 1 and 3 other SNPs on chromosome region 22 to be simulated as causal disease genetic variants. The simulated disease effect size parameter of those SNPs are summarized in the Table 2 below, and are chosen to fit small effect size in some studies and strong in other versus-versa.

MEX GWAS is considered to be our main study, and YRI and CEU GWAS are studies that we would like to pool information from in order to reveal larger effects in MEX GWAS and to provide valuable information that will be useful to prioritize the most important results.

3 SUPPLEMENTARY RESULTS

3.1 Text S3: Simulation and Evaluation results

3.1.1 Single-based GWAS results

To check the association signal from our simulated data sets, we conducted Genome-Wide Association Studies (GWAS) in each case-control dataset. To this end we used EMMAX Zhou and Stephens (2012). Table 3 displays the GWAS summary statistics of our simulated diseases SNPs. As expected, we obtain very weak signal of association from SNPs simulated with small effects, particularly form MEX and YRI. We obtained significant SNPs, particularly on chromosome 22 in CEU. Using a custom script in python, we plotted the Manhattan plots to represent the p-values of both chromosome 1 and 22 of GWAS on a genomic scale (Figure 1), for each simulated case-control data set.

3.1.2 Meta-analysis based on ancMETA at gene and sub-network levels

Here we used the GWAS summary statistics, consisting of study effect sizes, its standard errors and the associated p-values from the 3 simulated case-control data sets, to perform meta-analysis at gene and sub-network level using ancMETA. Further inputs required by ancMETA include (1) pairwise weighted Protein-Protein network. If Protein-Protein network is not weighted, ancMETA may use the genotype data of the study or a reference genotype data from the population close to those under study. This will allow ancMETA to compute the pair-wise SNP-LD and use it as described in the method to derive pairwise genes correlation coefficients. These pairwise gene correlation coefficients are used as the weight of the network. (2) Pathways dataset, that provides a list of pathways and associated genes.

Given these inputs, we conducted gene- and sub-network-specific meta-analysis under ancMETA. Table 4 displays top 4 genes with overall studies p-values $\leq 1.5e-08$ and the top 4 sub-networks represented by gene hubs.

The forest plot in Figure 2 indicates convergence of effect across studies, particularly simulated causal gene *CBX7*.

Importantly, the result in Table 4 indicates a convergence of the association signal in detecting true causal variants by leveraging not only multiple small effects at gene (or sub-network) level within a study, but also effects from cross studies. By leveraging the topological properties of network in significantly breaking into clustered sub-network, ancMETA identifies causal gene *CBX7* as central hub gene of one of the top significant sub-networks across these 3 studies.

3.2 Text S4: Application to Bipolar

3.2.1 Imputation fine-mapping on 7 European Bipolar Disorder GWAS data sets

As mentioned earlier, we obtained access to 7 European Bipolar Disorder GWAS cohorts (see Table 1).

Different GWAS on BD (Table 5) have been published in the last ten years, and have revealed interesting loci for understanding better the aetiology of this disorder. The general purpose of repeated GWAS was to discover genetic markers that are robustly associated with BD, to better characterize this complex disorder.

From each GWAS data set, we conducted LD-fine mapping base on the imputation of their GWAS summary statistics using the software ImpG-Summary (`http://bogdan.bioinformatics.ucla.edu/software/impg/`). ImpG-Summary implements a GWAS Gaussian imputation of summary statistics framework that leverage the haplotype and LD from close related reference haplotypes such as the $1,000$ Genomes. It is assumed that the summary statistics consist of Z-scores (note that they are distributed with mean 0 and the variance 1, under the null model of no association. The linkage disequilibrium between the marker i and j lead to a covariance among their observed Z-scores, due to the correlation r_{ij} between the two markers. In this way, under null data, the vector V of all Z-scores at all markers, is approximately distributed ($\sim \mathcal{N}(0, \Sigma)$) as the Gaussian distribution. We independently ran ImpG-Summary to each GWAS, resulting in millions of imputed z-scores of SNPs under LD with the original SNPs in the GWAS data sets.

For each population, Manhattan plots were created using a python script. Statistical strength of association (this means -log_{10} p-value) is plotted against genomic position with 22 chromosomes in different colors. The grey horizontal line indicates the genome-wide significance threshold of p-value = 5×10^{-08}. The vertical red-dotted points describe the highly significant SNPs associated with Bipolar disorder on a specific chromosome.

For Bipolar disorder GWAS on every population considered in this analysis framework except one population, we observed that the highly significant SNPs associated with Bipolar disorder are on chromosome 3. For the Scottish, British, Norwegian and European-American populations, the below Manhattan plots (see Figure S3) highlighted almost the same significant markers that are associated with Bipolar disorder, while we observed a different pattern of the distribution of significant SNPs associated with Bipolar disorder in the Irish population.

3.2.2 Gene- and sub-network-specific meta-analysis of 7 European GWAS summary statistics

Given the typed and the fine mapped GWAS summary statistics of these 7 European populations with Bipolar disorder, we conducted gene- and sub-network meta-analysis with relevant inputs required by ancMETA. At a gene level, 7 interesting genes were significant across these 7 studies (Table 7). Among these significant genes, *AGT* is strongly significant. These genes, are known to be associated with Bipolar disorder and other psychiatric disorders.

At sub-network level, we obtained 1 single significant sub-network of witch the gene *ESR1* is the central hub. This sub-network is well connected and includes several Bipolar disorder known associated genes and the most significant finding *AGT*

To further show the cross studies pattern of effect size and level of significance, We plotted the forest plots of these 7 genes (Figures 5-6).

4 SUPPLEMENTARY TABLES AND FIGURES

Tables captions

[0] BD-GWAS on European-American population* was done by Pritzker Neuropsychiatric Disorders Research Consortium, whereas BD-GWASs on European-American population** and European-American population*** were done by Systematic Treatment Enhancement Program for Bipolar Disorder into two steps.

Table S1. Study data description: For simulation based on 3 HapMap 3 populations and application of the tool based on 7 European Cohorts with Bipolar Disorder.

Population	Label	Cases	Controls	$Total sample$	# SNPs
Western Europe	CEU	0	165	165	1604948
Mexican ancestry	MEX	0	86	86	1604948
Yoruba	YRI	0	203	203	1604948
Irish population	IRI	150	797	947	617,245
Scottish population	SCT	282	275	557	332,354
European-American population*	EUA	1130	718	1848	317,213
European-American population**	EUA	922	645	1567	300,388
European-American population***	EUA	659	192	851	621,069
Norwegian population	NOR	203	349	552	504,025
British population	BRB	457	495	952	329,624

Table S2. The simulated disease effect size of simulated six SNPs. Het. risk (Heterozygosity risk) and Homo. risk (Homozygosity risk)

CHR	SNPs	Gene	**CEU**			**YRI**			**MEX**		
			**Het risks**	**Homo. risk**	**risk allele**	**Het. risks**	**Homo risk**	**Risk allele**	**Het risk**	**Homo risk**	**Risk allele**
1	*rs1204389*	*LOC105373298*	3.5	2.25	1	3.5	2.25	0	1.1	0.98	1
1	*rs3128291*	*PRKCZ*	4.5	3.2	0	2.5	2.25	1	1.0	1.6	1
1	*rs11590989*	*ASTN1*	5.5	5.2	1	1.0	1.25	0	1.2	1.8	0
22	*rs131154*	*RTL6*	2.95	6.1	0	4.2	3.25	0	2.2	1.8	0
22	*rs17002000*	*MKL1*	2.95	6.1	2.0	4.2	1.25	2.1	1.6	1.8	1.0
22	*rs139418*	*CBX7*	2.95	7.1	2.1	2.2	1.2	2.1	1.7	1.88	1.2

Table S3. Single-SNP GWAS results from case-control simulated datasets: Displaying the signal of simulated variants.

			**CEU**		**YRI**		**MEX**	
SNP	CHR	Closest Gene	P	$Beta \pm SD$	P	$Beta \pm SD$	P	$Beta \pm SD$
rs3128291	1	*PRKCZ*	0.034	0.022 ± 0.0105	0.263	-0.024 ± 0.021	0.1814	0.057 ± 0.043
rs131154	22	*RTL6*	0.0001	0.0408 ± 0.0107	0.09	-0.0031 ± 0.018	0.061	-0.059 ± 0.031
rs139418	22	*CBX7*	$4.33e-14$	-0.0105 ± 0.0013	$4.18e-07$	0.055 ± 0.010	0.016	-0.028 ± 0.012
rs1204389	1	*LOC105373298*	0.86	-0.026 ± 0.15	0.30	0.025 ± 0.025	0.59	0.028 ± 0.053
rs17002000	22	*MKL1*	$1.37e-10$	-0.0105 ± 0.0016	$9.25e-05$	0.055 ± 0.014	0.018	-0.028 ± 0.012

Table S4. Meta-Analysis at gene and sub-network level:

					Study P-values			Study M-values		
**Genes**	**#Study**	**P**	**Q**	**Tau Square**	**MEX**	**YRI**	**CEU**	**MEX**	**YRI**	**CEU**
LDOC1L	3	1.33e-10	2.055	0.0007	0.012	0.00019	0.0003	0.98	0.91	0.81
PRKCZ	3	1.77e-11	2.193	0.001	0.001	0.047	0.001	0.40	0.41	0.41
CBX7	3	0.00000001	1.825	0	0.0001	0.001	0.0001	0.90	0.91	0.90
ASTN1	3	2.006e-8	2.261	0.001	0.0012	0.001	0.0006	0.81	0.91	0.91
**Hub Sub-network**	**#Study**	**P**	**Q**	**Tau Square**	**MEX**	**YRI**	**CEU**	**MEX**	**YRI**	**CEU**
HNRNPA1	3	0.00000003	2.09	0.00008	0.09	0.048	0.03	0.59	0.59	0.59
PRKCZ	3	1.93e-7	2.2	0.0002	0.019	0.04	0.021	0.59371	0.59	0.59
CBX7	3	4.25e-7	2.1	0.0002	0.010	0.021	0.044	0.60	0.59	0.59
KRT18	3	0.0000004	1.90	0	0.049	0.09	0.005	0.59	0.59	0.59

Authors	Year	Discovery population	Validation population	Cases (male; Female)	Control (male; female)	SNP	Genes of interest	Chromosome /Locus	p value
WTCCC	2007	UK	-	1868	2938	rs420259	PALP2/NDUFAB1/DCTN5	$16P12$	6.3×10^{-8}
Sklar et al.	2008	US/Uk	Caucasian/US	1461	2008	rs4939921	MYO5B	18q21	1.66×10^{-7}
						rs1705236	TSPAN8	12q14.1-q21.1	6.11×10^{-7}
Ferreira et al.	2008	European/US	European/US	4387	6209	rs10994336	ANK3	10q21	9.1×10^{-9}
						rs1006737	CACNA 1C	12p13.3	7.0×10^{-8}
Baum et al.	2008 a	European	German/US	1233	1439	rs1012053	DGKH	$13q14.11$	1.5×10^{-8}
Baum et al.	2008 b	European	UK/German/US	3101	4377	rs4806874 rs10791345	SLC39A3/JAM3	$19p13.3/11q25$	$9 \times 10^{-6}/5 \times 10^{-6}$
ISC	2009	European	European European-American African-American	3322(2176M; 1146F)	3587 (1642M; 1945F)	rs5761163	$MYO18B$	$22q11.2$	3.4×10^{-7}
Hattori et al.	2009	Japanese	Japanese	107 (35M; 54F)	107 (35M; 54F)	rs6540451	PLXNA2	$1q32.2$	7.5×10^{-3}
Scott et al.	2009	European	European ancestry	2076	1676	rs1042779	$mH1$	3	1.8×10^{-7}
Smith et al.	2009	US/Uk	European/African	$1001(E)/345(A)$	$1033(E)/670(A)$	rs5907577 rs10193871 (E)/rs2111504; rs2769605; (A)	Intergenic/NAP5 (E)/DPY19L3/NTRK2(A)	$Xq27.1/2q21.2$ $(E)/19q13.11/9q21.33$ (A)	$1.6 \times 10^{-6}/9.8 \times 10^{-6}$ $(E)/1.5 \times 10^{-6}/4.5 \times 10^{-5}$ (A)
McMahon et al.	2010	US/UK/European	European	6683	9068	rs2251219	Polybromo-1(PBRM1)	$3p21$	1.7×10^{-9}
Djurovic	2010	Norwegian	Icelander	194	336	rs1750565, rs1798968 rs1750567, rs11617400	DLEU2/GUCY1B2	$13q14.2 - 14.3$	$2.4 \times 10^{-5}/3.7 \times 10^{-5}$
Lee et al.	2010	Han Chinese	Han Chinese	1000	1000	rs2709736 rs8040009 rs2073831 rs11013860	SP8/ST8SIA2/KCTD 12/ CACNB2	$7p21/15q26/13q22.3/$ $10p12$	$4.9 \times 10^{-7}/6.1 \times 10^{-6}/$ $9.7 \times 10^{-6}/5.2 \times 10^{-5}$
Belmonte Mahon et al.	2011	US/European	US/European	2836	2744	rs16883399, rs455219, rs10267593, rs2623968, rs17054536	MBOAT1/FAT1/MAD 1L1/ SYNE1/ECHDC1	chromosome $6/4/7/6/6$	$6.8 \times 10^{-5}/1.5 \times 10^{-4}$ $2.3 \times 10^{-4}/9.7 \times 10^{-4}/$ 1.9×10^{-4}
Cichon et al.	2011	European	European	2411	3613	rs1064395	NCAN	$19p13.11$	3.0×10^{-8}

Table S5. Different published GWAS on Bipolar Disorder. WTCCC stands for to The Wellcome Trust Case Control Consortium.

Table S6. Gene and sub-network-specific meta-analysis on 7 European populations with Bipolar disorder from GWAS summary statistics.

				Study Pvalues							M-values						
**Gene**	**#Study**	**Overal P**	**Tau Square**	**IRI**	**NOR**	**EUA**	**EUA**	**BRB**	**EUA**	**SCT**	**IRI**	**NOR**	**EUA**	**EUA**	**BRB**	**EUA**	**SCT**
AGT	7	3.20e-19	0	3.21e-7	4.82e-8	2.072e-5	5.29e-9	6.03e-6	3.54e-6	6.54e-9	0.9345	0.95	0.81	0.95	0.89	0.89	0.99
CACNA1C	7	1.10e-5	0	0.017	0.008	0.002	0.003	0.015	0.001	0.037	0.39	0.39	0.37	0.35	0.36	0.39	0.43
NCAN	7	9.00e-3	0	0.003	0.003	0.015	0.012	0.015	0.003	0.002	0.63	0.53	0.43	0.47	0.39	0.4	0.53
ESR1	7	3.38e-4	0	0.009	0.001	0.001	0.006	0.002	0.002	0.006	0.38	0.43	0.38	0.53	0.48	0.41	0.63
BDNF	7	5.00e-3	0.0025	0.015	0.002	0.006	0.001	0.006	0.002	0.002	0.39	0.39	0.38	0.43	0.43	0.73	0.43
BCR	7	2.40e-3	0	0.002	0.002	0.006	0.001	0.001	0.004	0.009	0.38	0.36	0.43	0.33	0.63	0.53	0.4
GSK3B	7	2.70e-3	0.055	0.005	0.004	0.018	0.001	0.024	0.0009	0.002	0.33	0.33	0.341	0.33	0.34	0.33	0.33
**Hub sub-network**	**#Study**	**Overal P**	**Tau Square**	**IRI**	**NOR**	**EUA**	**EUA**	**BRB**	**EUA**	**SCT**	**IRI**	**NOR**	**EUA**	**EUA**	**BRB**	**EUA**	**SCT**
ESR1	7	1.032e-12	0	3.20e-7	4.82e-8	2.072e-5	5.29e-9	6.039e-6	3.5e-6	6.54e-9	0.93	0.95	0.81	0.95	0.89	0.89	0.99

Figures captions

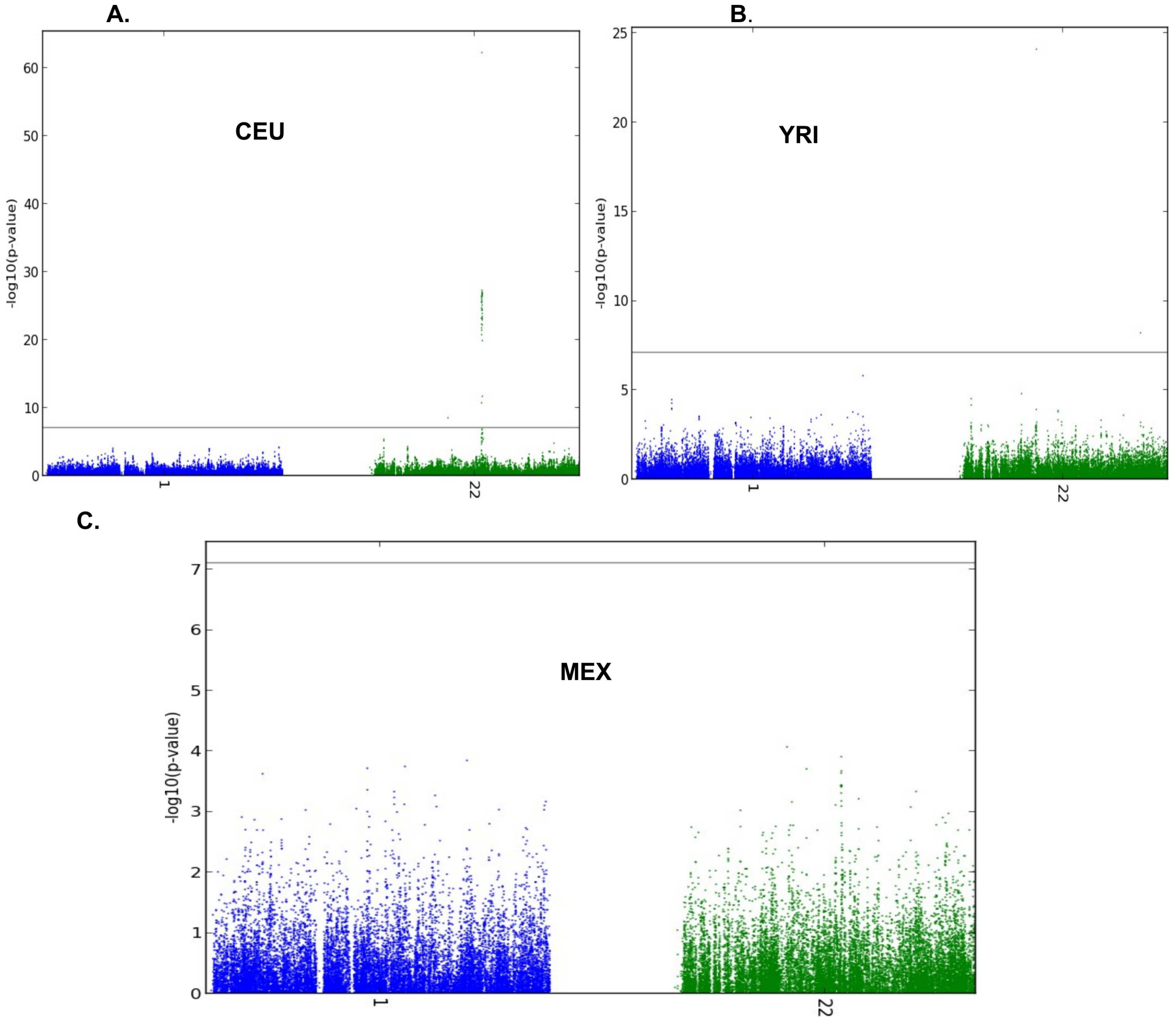

Figure S1. Manhattan plots represent the p-values of GWAS on a genomic scale based chromosome 1 and 22 for the simulated case-control CEU, YRI and MEX.

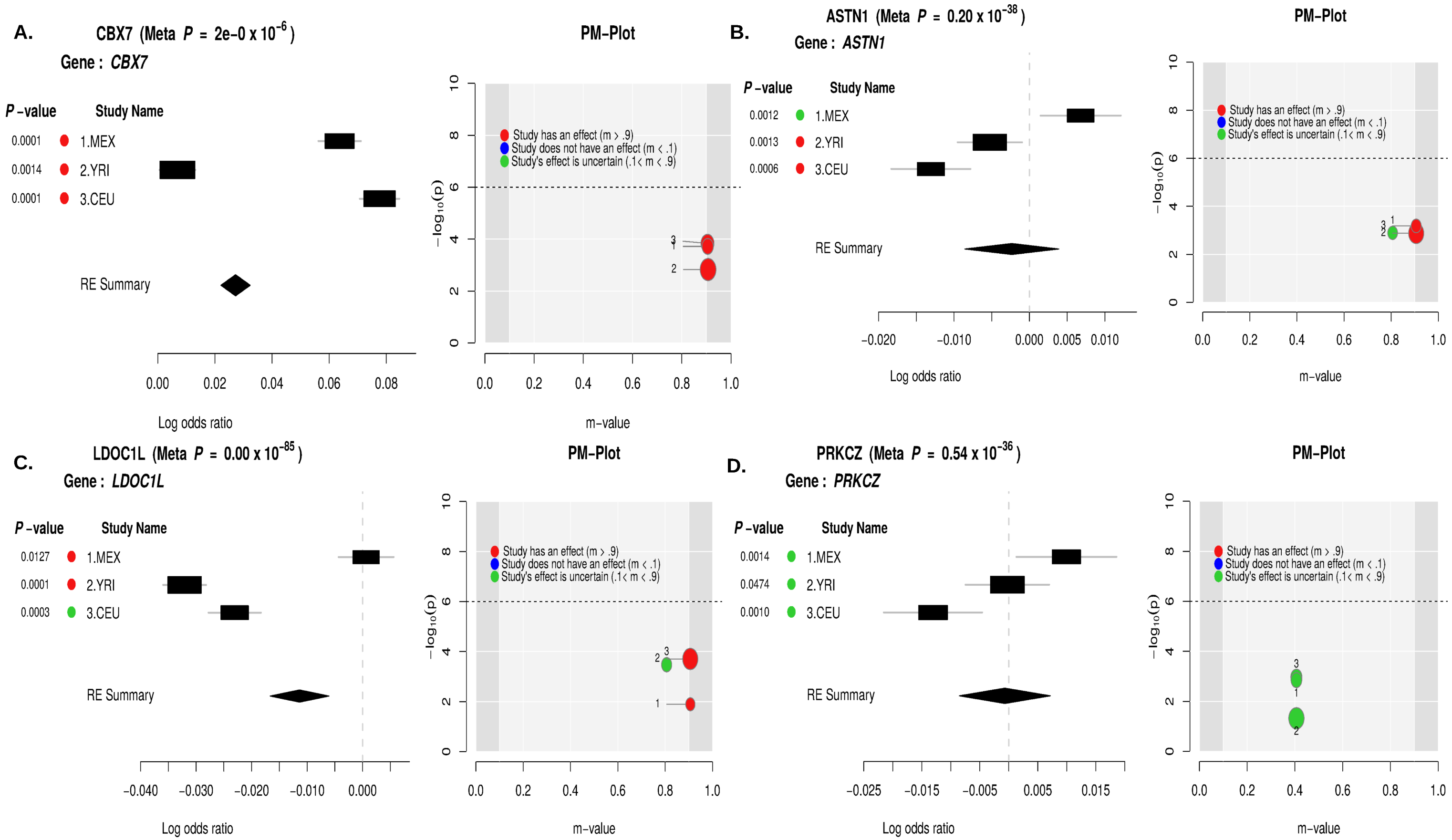

Figure S2. Forest plot of the top 4 significant genes from ancMETA, meta-analysis at a gene level across three simulated case-control data sets, CEU, YRI and MEX.

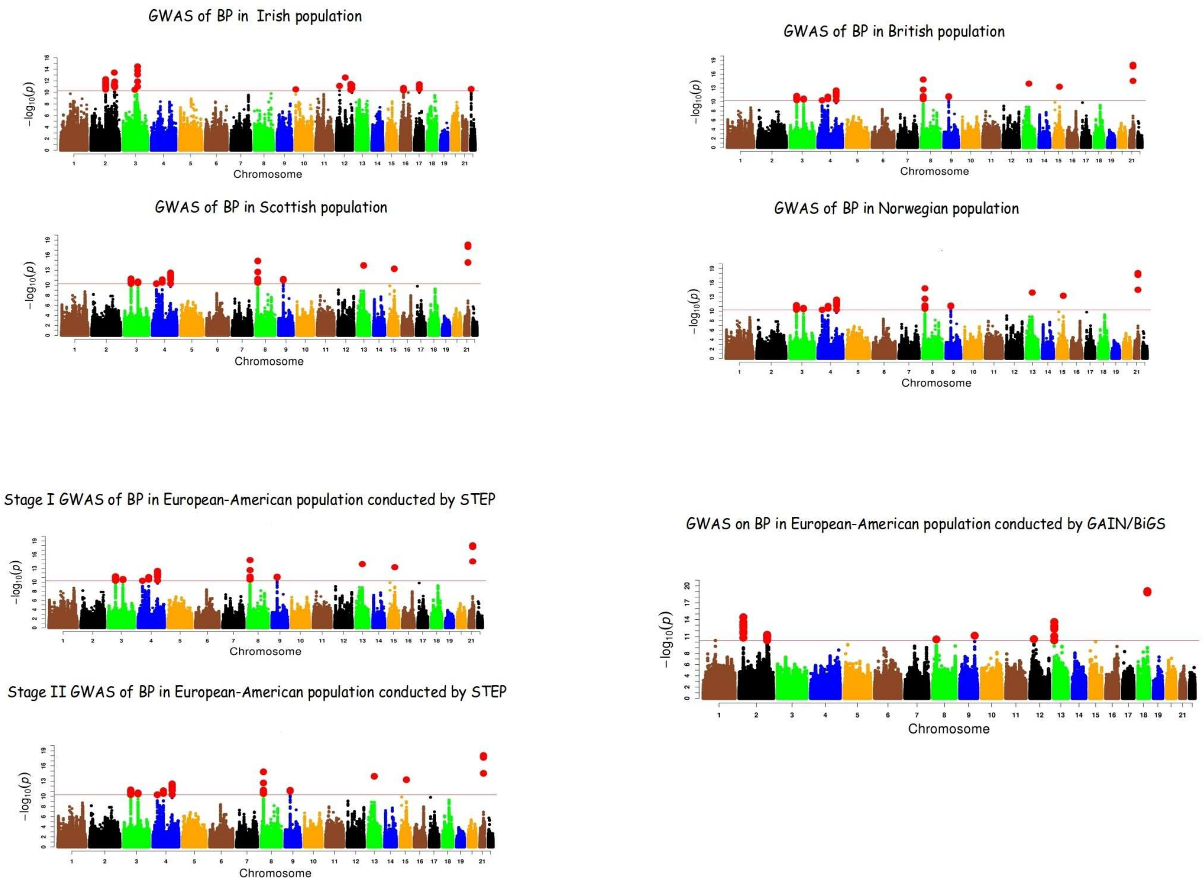

Figure S3. Manhattan plots for GWAS-replicate on Bipolar (BP) in different European populations.

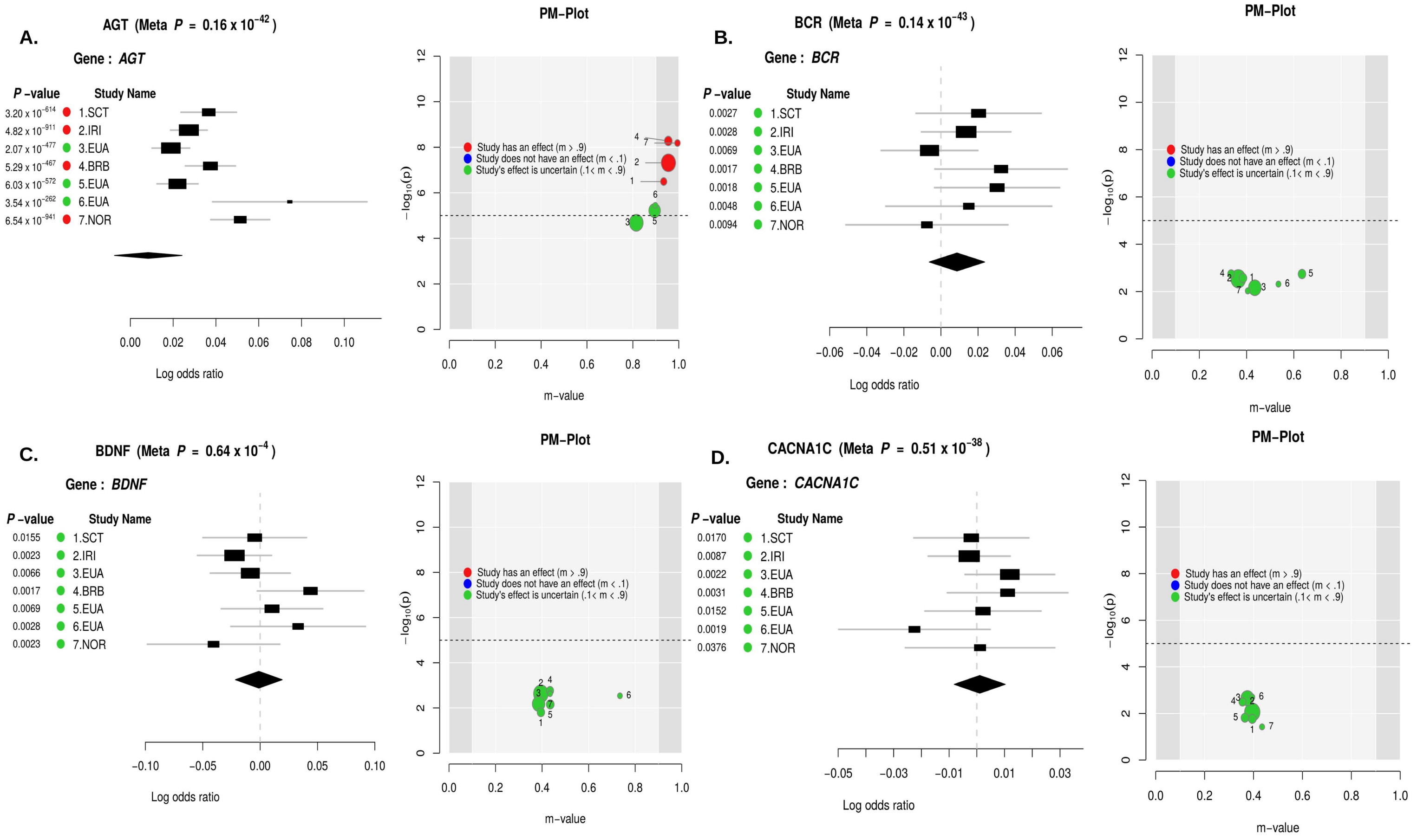

Figure S4. Forest plot of top 7 significant genes from ancMETA, meta-analysis at gene level across 7 European populations with Bipolar disorder from GWAS data sets.

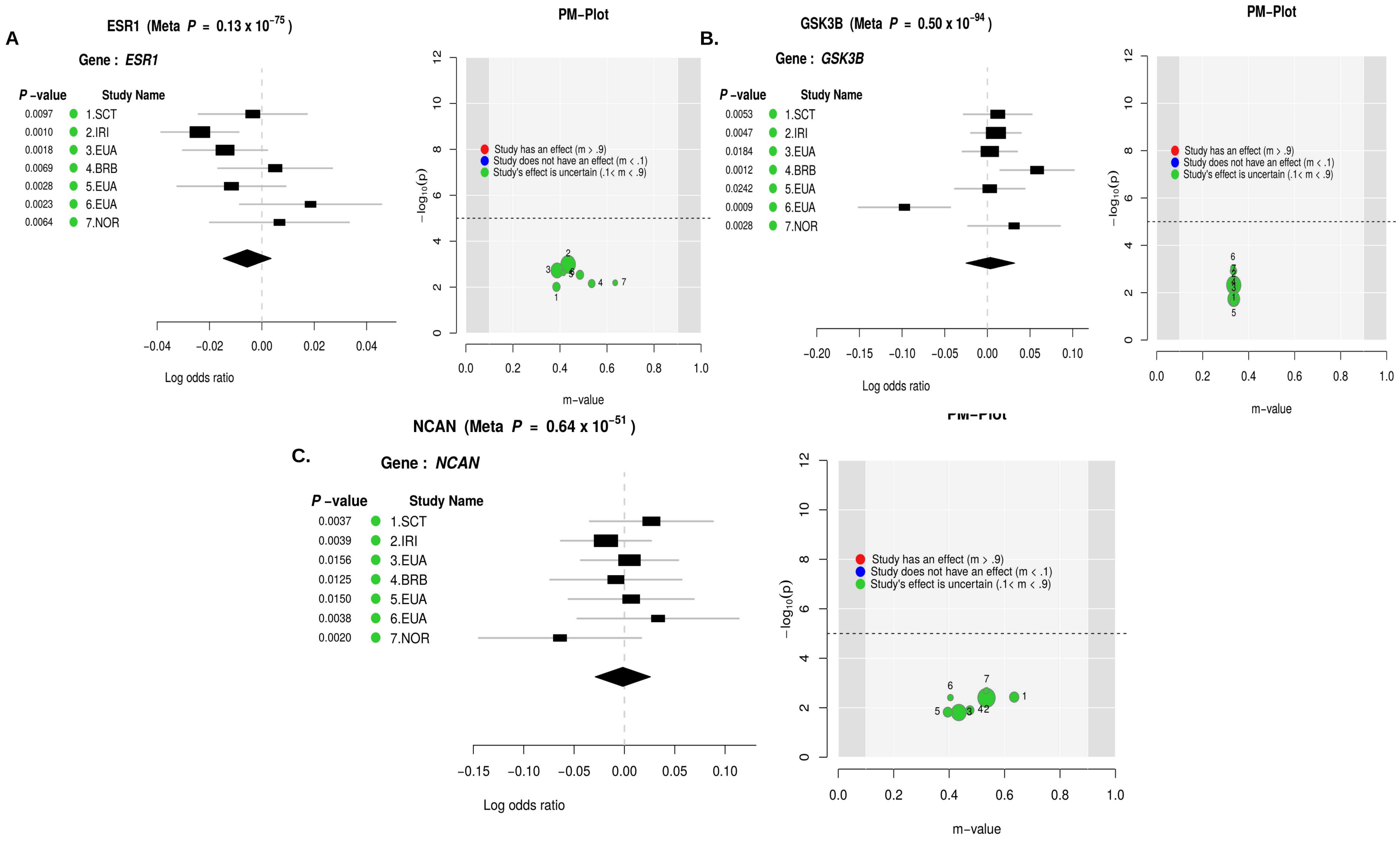

Figure S5. (continue))Forest plot of top 7 significant genes from ancMETA, meta-analysis at a gene level across 7 European populations with Bipolar disorder from GWAS data sets.

REFERENCES

Benjamini, Y. and Hochberg, Y. (1995). Controlling the false discovery rate a practical and powerful approach to multiple testing. *J. Royal Stat. Soc. Series B-Methodol., 57, 289-300*

Berger, S., Posner, J., and Maayan, A. (2007). Genes2networks: connecting lists of gene symbols using mammalian protein interactions databases. *BMC bioinformatics 8: 372*

Chimusa, E., Mbiyavanga, M., Mazandu, G., and Mulder, N. (2015). ancgwas: a post genome-wide association study method for interaction, pathway and ancestry analysis in homogeneous and admixed populations. *Bioinformatics, 32(4), pp.549-556*

Choi, S. (1977). Tests of equality of dependent correlation coefficients. *Biometrika 64 (3): 645–647. doi:10.1093/biomet/64.3.645*

Frazer, K. and et al (2007). A second generation human haplotype map of over 3.1 million snps. *Nature. 449, 851-861*

Kerrien, S., Aranda, B., Breuza, L., Bridge, A., Broackes-Carter, F., Chen, C., et al. (2012). The intact molecular interaction database in 2012. *Nucleic Acids Research;40(Database Issue):D841–D846*

Kristin, C., Kruglyak, L., and Seielstad, M. (2002). Patterns of linkage disequilibrium in the human genome. *Nature Reviews Genet. 3, 299-309*

Li, N. and Stephens, M. (2003). Modeling linkage disequilibrium and identifying recombination hotspots using single-nucleotide polymorphism data. *Genetics, 165(4), 2213–2233. https://doi.org/10.1093/genetics/165.4.2213*

Loh, P., Danecek, P., Palamara, P., Fuchsberger, C., Reshef, Y., Finucane, H., et al. (2016). Reference-based phasing using the haplotype reference consortium panel. *Nature genetics, 48(11), pp.1443-1448*

Mazandu, G. and Mulder, N. (2011). Generation and analysis of large-scale data-driven mycobacterium tuberculosis functional networks for drug target identification. *Adv Bioinformatics:ID 801478, 14*

McVean, G., Altshuler, D., Durbin, R., Abecasis, G., Bentley, D., Chakravarti, A., et al. (2012). An integrated map of genetic variation from 1,092 human genomes. *Nat. 491 (7422), 56-65*

Orchard, S., Ammari, M., Aranda, B., Breuza, L., Briganti, L., Broackes-Carter, F., et al. (2014). The mintact project - intact as a common curation platform for 11 molecular interaction databases. *Nucleic Acids Res. 2014, 42, D358–D363*

Su, Z., Marchini, J., and Donnelly, P. (2011). Hapgen2: simulation of multiple disease snps. *Bioinformatics. 7 (16), 2304-2305*

Williams, N. (1997). *Bayesian Learning for Neural Networks*

Zhang, F., Guo, X., and Ma, J. (2012). Pathsimu: A flexible simulating tool for pathway-based genome-wide association studies. *1: 116. doi:10.4172/scientificreports.116*

Zhou, X. and Stephens, M. (2012). Genome-wide efficient mixed-model analysis for association studies. *Nat Genet. 44(7), 821-4*
